# Deadly Mass Shootings, Mental Health, and Policies and Regulations: What We Are Obligated to Do!

**DOI:** 10.3389/fped.2018.00099

**Published:** 2018-04-16

**Authors:** Marie Leiner, Izul De la Vega, Bert Johansson

**Affiliations:** ^1^Department of Pediatrics, Texas Tech University Health Sciences Center, El Paso, TX, United States; ^2^Political Science, UCLA Department of Political Science, Los Angeles, CA, United States

**Keywords:** mental health prevention, young adult, communication barriers, child, schools

Conversations about mass shootings in the United States, particularly school shootings, should not be a temporary reflection. Rather, these shootings should prompt continuous action from citizens until something effective is done. It is particularly important to stop repeating past errors, such as focusing on who, or what, to blame (1). Most likely, the impetus behind the shootings cannot be found in a singular cause but is instead an intersection of many issues: mental health problems, a culture of violence, gun regulations, the consequences of poverty, etc. Perhaps the most pressing issue is the inability to establish a dialog between all involved parties to find reasonable solutions.

## A Call for Action

The most recent mass shooting at Parkland High School resulted in over a dozen murdered students and a multitude of emotionally and physically scarred survivors. Their families, friends, and classmates will likely face lifelong consequences by carrying short- and long-term memories of devastation, violence, and suffering, simply because as a society we have not done enough to stop mass shootings. Perhaps, the latest victims’ responses can inspire us, independent of our own political, sociological, or financial interests, to join their call for action. By joining them, we can finally meet our obligations as a society. Lorrie Alhadeff, the mother of Alyssa Alhadeff, murdered during the shooting kept calling for “action, action, action” when addressing the President of the United States. Her voice, along with the voices of other victims, should not be limited to the news cycle that immediately follows the shooting, but should have a long-lasting influence on policy. The Z generation is setting an example by fiercely confronting the issue. In response, we as academicians, students, and citizens should support their demands for a dialog that is long overdue.

## Mental Health Issues

Mental health is a topic that necessitates more attention (not exclusively) considering that prevention should be the base of the pyramid of strategies. Funding for programs cannot be limited to care, but should also focus on prevention, and programs for young people already suffering from mental illness. In regard to care, there is a potentially devastating gap in mental health services in the United States. Millions of adults have behavioral conditions, including at least three million with serious mental health conditions that are not receiving treatment (2). In 2015, 63% of ~34 million adults with mild and moderate conditions were unattended and 89% of ~20 million adults needed substance abuse treatment. Services provided to these adults indicate a lack of services in the previous year with only 20% adults (any mental health conditions), 40% (serious mental health condition), and 90% (substance use services) receiving care.

Many of these individuals lack options for care and attend emergency services accounting for 3–4% of all emergency services provided. To blame violent acts on mental health problems might be inaccurate. The reality is that only 3–5% of all violent acts are committed by people with severe mental illness. Moreover, individuals with mental health problems are 10 times more likely to be victims of a violent crime when compared with the general population (3). However, many people with mental health problems are currently incarcerated. Prisons have increasingly become the nation’s mental health treatment facilities holding individuals accused of lower level crimes such as trespassing, disorderly conduct, or theft (4). Along with limited options for care, there is a scarcity of programs that take preventative measures toward individuals where disparities most likely contribute to mental or behavioral issues.

## Preventing Mental Health Issues at an Early Age

Aggressive behaviors among children are becoming an increasingly important and challenging topic in mental health care. These behaviors tend to start in early childhood and have been observed in up to 72% of children from ages 12–16 (5). Evidence suggests that should these aggressive behaviors become norms, the risk for serious problems, including school failure, drug addiction, and early pregnancies in adolescence, is three times higher than for individuals who possess better coping strategies (6). Especially at risk for these negative outcomes are children who belong to lower socioeconomic groups, who have teenage and/or single parents, or who demonstrate a difficult temperament (7). It appears that growing up in poverty can especially exacerbate these major behavioral problems and can be part of a cycle in which poverty contributes to mental illness while mental illness reinforces poverty (8). Lower socioeconomic status also reduces the opportunity for early intervention as it negatively affects access to health care or to mental health services, thus limiting detection, referral, and treatment opportunities (9). Other barriers such as language and literacy levels also contribute to these disparities (10–13).

Published evidence suggests that reducing aggressive behaviors in young children is more effective than later interventions and may prevent the development of aggression as the preferred form of interaction with others (14). It is thus important to intervene early to reduce or avoid more serious behavioral problems later. Programs directed at parenting, as well as those that increase social and communication skills among pre-school children will have a high impact on their mental wellbeing later on in life.

The future for young people, where inaction continuously follows mass shootings, looks bleak without a concerted effort to address the factors that may contribute to mass shootings. Schools have traditionally been viewed as safe places, but now students are required to run drills in preparation for an active shooter situation. Many families live in a culture of violence, either in media or real life, but those who are confronted with disparities have a higher risk of suffering behavioral, social, and mental health problems. Young people are cycling between bullies, bully-victims, or victims and need programs that help them to move out of this process. Investment in preventative programs can reduce the production of mental health problems. It is important to consider a comprehensive approach that both improves school safety (15) and cultivates the conditions that promote equity among school-age children. School shootings are overwhelmingly carried out by disaffected and angry youths (16).

## Overview of School Shootings

An analysis of 175 years of school shootings (1840–2015) including 304 events identifies as the primary factor for most shootings 185 (61.0%) as “anger,” “fight,” and “dispute” (combined). Secondary factors from those 185 events include [25 (14%) related to discipline, 19 (10%) related to harassment, 18 (10%) to dismissal (or failure or a bad grade), 14 (8%) to revenge, 7 (4%) to romance, and 4 (2%) to some domestic issue (i.e., domestic abuse or some other domestic issue) (2%)] (16). The same study includes 20 mass murder events (where at least 4 people died), where 6% of the shooting events accounting for 43% of the deaths and 37% of the injuries. From 1840 until 1966, only three mass murder events occurred at an educational institution (14 deaths and 4 injuries). After 1966, 17 events have resulted in 166 deaths and 204 injuries accounting for 85% of the mass murder shooting events since 1966, 92% of the deaths and 98% of the injuries.

## Reframing the Debate

Due to severity of these numbers, it is important to reframe the debate on gun regulations as a potential win–win situation for all interested parties. While it is possible that both sides have valid points, neither side is being heard. It is possible the scarcity of debates on gun control stems from the financial support they provide to political candidates as an incentive for turning the conversation away from gun control. According to a study from the *Center for Responsible Politics*, a nonpartisan think tank, the National Rifle Association funneled $5.9 million into Republican candidates during the 2016 election cycle. Nonetheless, we must acknowledge that the causes for gun violence are an intersection of many problems and we need to turn our focus toward other methods to reduce gun violence. Author Pamela Haag, in her book, *The Gunning of America*, claims that gun companies not only manufacture guns but also manufactured the demand for them. She dismisses the idea that America has a gun culture but instead the culture was created by corporations and given to us (1). At the very least, these marketing strategies are tangentially responsible for providing the guns that have fueled the US homicide rate to 20 times higher than the combined rates of 22 similarly developed countries.

The right to bear arms is not the issue that needs to be confronted, but rather the regulations that need to be discussed for the benefit of all. It is important to note that since the massacre in Newtown over 100 pieces of gun legislation have failed in Congress and not even one reform has passed. It is an unfortunate measure of our society that the shooting deaths of over a dozen first-graders was met with empty thoughts and prayers and little deliberate action to prevent a massacre in the future.

## Conclusion

It is unclear if only regulations can resolve this complex problem (17) instead it is necessary to have a dialog where all interested parties have a voice and an interest to solve the problem using a holistic approach. The best approach should place responsibility where it belongs and should not place the interests of a few over the benefit of all. We need to support the movement that has emerged from the aftermath of the Parkland High School shooting and actively pursue solutions to mass shootings, regardless of ideology, profession, or financial interest. This might include a multidisciplinary approach to the problem that brings different issues to the debate, such as mental health services and mental health prevention funding, programs to increase access to resources for at risk youth, and a reasonable approach to gun control. Let us not to forget that we are obligated as society to provide our youth with positive role models, a sense of safety, and hope for the future.

## Author Contributions

ML, IV, and BJ make substantial contributions to conception and design of draft a final paper, based on their professional experience.

## Conflict of Interest Statement

The authors declare that the research was conducted in the absence of any commercial or financial relationships that could be construed as a potential conflict of interest.
